# Supplementary material for: Cyclin-dependent Kinase 1 and Aurora Kinase choreograph mitotic storage and redistribution of a growth factor receptor
Source: PLoS Biol. 2021 Jan 4;19(1):e3001029. doi: 10.1371/journal.pbio.3001029 (PMC7808676; doi:10.1371/journal.pbio.3001029)

S1 Fig. Inhibition of mitotic entry suppresses FGFR mitotic trafficking but does not impact TVC induction (Related to Fig 1). (**A-B'**) Ventral projections and lateral sections for founder cells electroporated as indicated. Dashed lines (**A&B**; orange) indicate position of sections (**A'&B'**). (**C**) Graphical summary of regional FGFR::VENUS enrichment for founder cells electroporated as indicated. No significant changes in regional FGFR::VENUS enrichment were detected in arrested *Mesp>Cdki(p27)* transgenic founder cells (plasma membrane-associated p=0.489, peripheral cytoplasm p=0.527, deep cytoplasm p=0.899). Data were obtained from 2 independent trials, n>16. (**D**) Graphical summary of mitotic arrest at different stages as observed for founder cells electroporated with either *Mesp>LacZ* or *Mesp>Cdk1(p27)* as indicated. Data were obtained from 3 independent trials, n>13 per trial. (**E-F''**) Representative micrographs of late tailbud embryos showing cranial-cardiac progenitor induction (indicated by overlaping *Mesp>Ensc::GFP* and *FoxF>RFP* reporter expression) versus non-induced pre-cardiac founder lineage cells (indicated by *Mesp> Ensc::GFP* reporter expression alone) in embryos co-electroporated with either *Mesp>LacZ* or *Mesp>Cdk1(p27)* as indicated [20,40,41,21]. ATM=Anterior Tail Muscle Cell, TVC = cranial-cardiac progenitor/trunk ventral cell. (**G-H**) Graphical summary of mitotic arrest and heart progenitor induction in embryos co-transfected as indicated. Data were obtained from 3 independent trials, n>17 per trial. Scale bars are indicated in micrometers. Significance indicated; n.s.= not significant. Significance was determined using one-way ANOVA followed by Tukey's multiple comparison test. Error bars represent S.E.M. Numerical values for all graphs can be found in S6 Data.

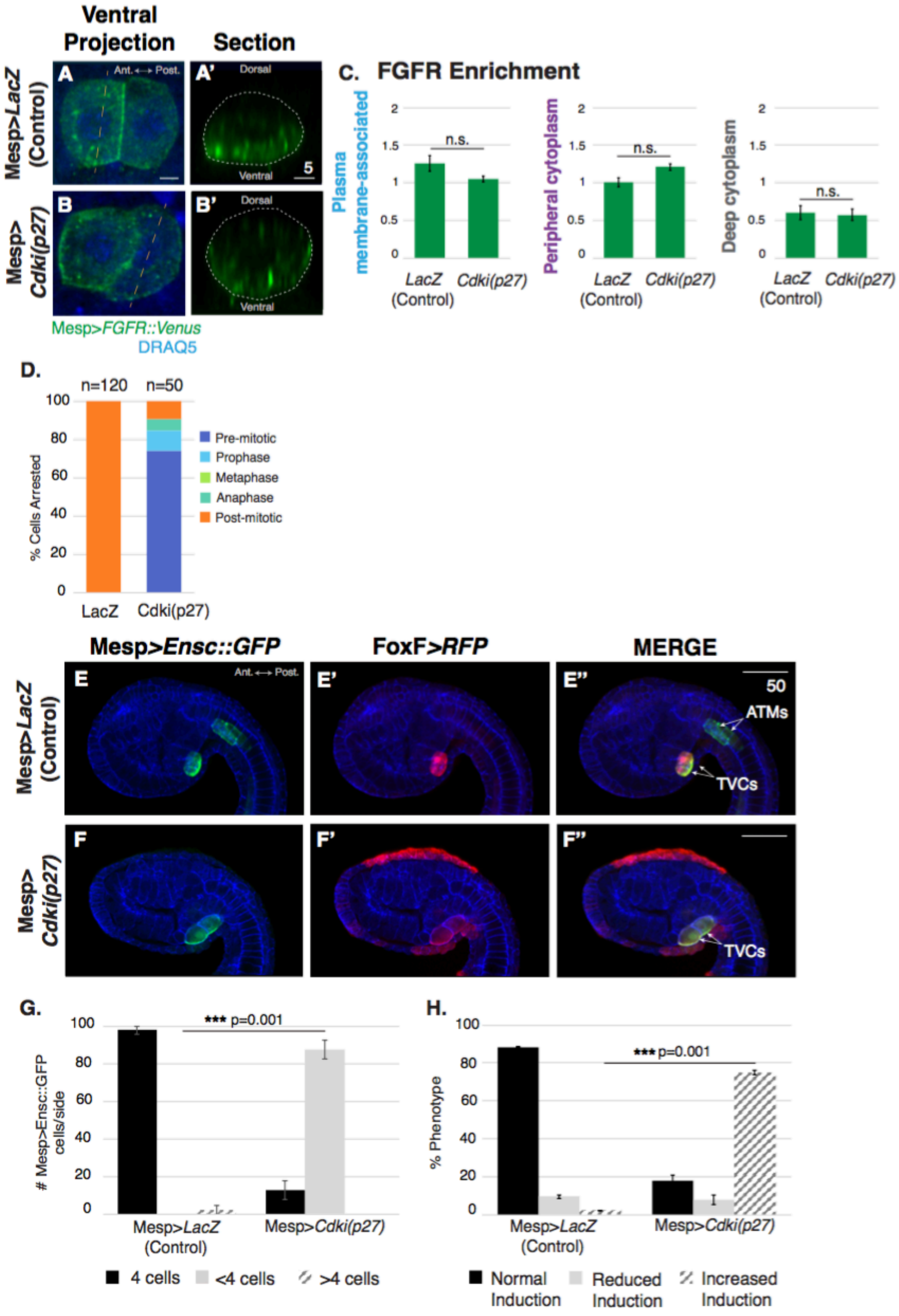

Supplement: S1 Fig — (A-B’) Ventral projections and lateral sections for founder cells electroporated as indicated. Dashed lines (A and B; orange) indicate position of sections (A’ and B’). (C) Graphical summary of regional FGFR::VENUS enrichment for founder cells electroporated as indicated. No significant changes in regional FGFR::VENUS enrichment were detected in arrested Mesp>Cdki(p27) transgenic founder cells (plasma membrane-associated p = 0.489, peripheral cytoplasm p = 0.527, deep cytoplasm p = 0.899). Data were obtained from 2 independent trials, n > 16. (D) Graphical summary of mitotic arrest at different stages as observed for founder cells electroporated with either Mesp>LacZ or Mesp>Cdk1(p27) as indicated. Data were obtained from 3 independent trials, n > 13 per trial. (E-F”) Representative micrographs of late tailbud embryos showing cranial-cardiac progenitor induction (indicated by overlapping Mesp>Ensc::GFP and FoxF>RFP reporter expression) versus noninduced precardiac founder lineage cells (indicated by Mesp>Ensc::GFP reporter expression alone) in embryos coelectroporated with either Mesp>LacZ or Mesp>Cdk1(p27) as indicated [20,40,41,21]. (G-H) Graphical summary of mitotic arrest and heart progenitor induction in embryos cotransfected as indicated. Data were obtained from 3 independent trials, n > 17 per trial. Scale bars are indicated in micrometers. Significance indicated; n.s., not significant. Significance was determined using one-way ANOVA followed by Tukey multiple comparison test. Error bars represent SEM. Numerical values for all graphs can be found in S6 Data. ATM, Anterior Tail Muscle Cell; FGFR, Fibroblast Growth Factor Receptor; SEM, standard error of mean; TVC, Trunk ventral cell/Cranial-cardiac progenitor. (PDF) [file pbio.3001029.s001.pdf]
