# Supplementary material for: Cyclin-dependent Kinase 1 and Aurora Kinase choreograph mitotic storage and redistribution of a growth factor receptor
Source: PLoS Biol. 2021 Jan 4;19(1):e3001029. doi: 10.1371/journal.pbio.3001029 (PMC7808676; doi:10.1371/journal.pbio.3001029)
Supplement: S2 Fig — (A-A”’) Graphical summary of whole cell (A) and regional FGFR::VENUS/ CLIP::RAB7 colocalization (A’-A”’; Manders’ overlap) during founder cell division (data shown correspond to data presented in Fig 2D). n > 6 for each mitotic stage. Regional overlap was measured in 3 concentric regions, plasma membrane, peripheral cytoplasm, and deep cytoplasm (Fig 1A-A”; Methods). Lack of any significant change (p > 0.05) is indicated by no change in lettering (a for all columns). Significance was determined using one-way ANOVA followed by Tukey multiple comparison test. Numerical values for all graphs can be found in S7 Data. Error bars represent SEM. FGFR, Fibroblast Growth Factor Receptor; SEM, standard error of mean. (PDF) [file pbio.3001029.s002.pdf]

S2 Fig

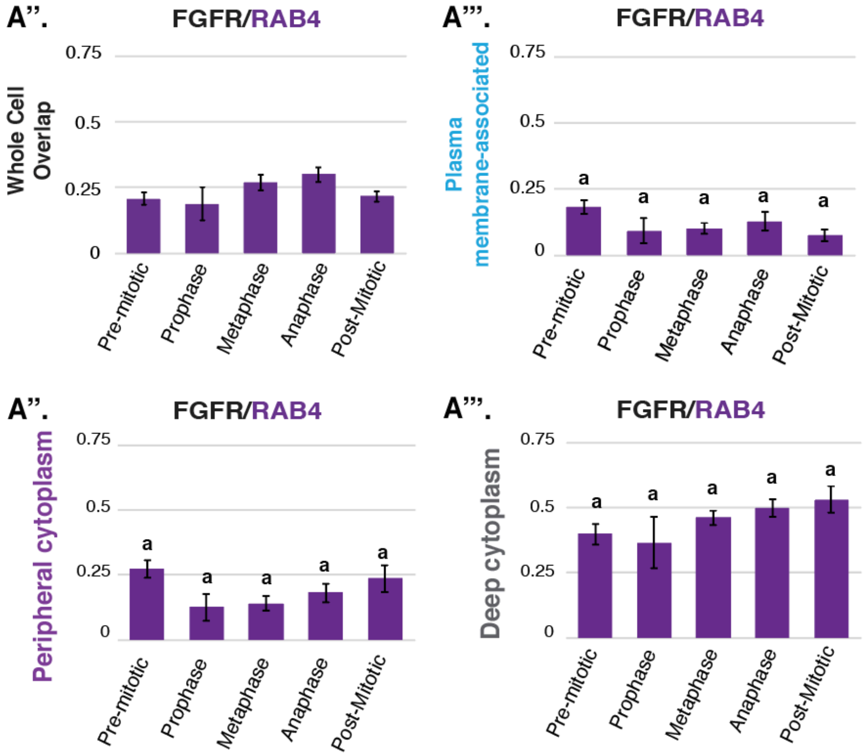

S2. Stage-specific quantitation of mitotic FGFR trafficking patterns (Related to Figure 2). (**A-A'''**) Graphical summary of whole cell (**A**) and regional FGFR::VENUS/ CLIP::RAB7 colocalization (**A'-A'''**; Manders' overlap) during founder cell division (**data shown corresponds to data presented in Figure 2D**). n>6 for each mitotic stage. Regional overlap was measured in three concentric regions, plasma membrane, peripheral cytoplasm and deep cytoplasm (**Figure 1A-A''**; Methods). Lack of any significant change (p>0.05) is indicated by no change in lettering (a for all columns). Significance was determined using one-way ANOVA followed by Tukey's multiple comparison test. Numerical values for all graphs can be found in S7 Data. Error bars represent S.E.M.
