# Supplementary material for: Cyclin-dependent Kinase 1 and Aurora Kinase choreograph mitotic storage and redistribution of a growth factor receptor
Source: PLoS Biol. 2021 Jan 4;19(1):e3001029. doi: 10.1371/journal.pbio.3001029 (PMC7808676; doi:10.1371/journal.pbio.3001029)
Supplement: S3 Fig — (A-B’) Masked/thresholded transverse sections of founder cells electroporated with Mesp>FGFR::Venus and Mesp>HALO::RAB11 and treated as indicated. For clarity, panels showing only colocalized FGFR::VENUS/CLIP::RAB11 puncta are provided (OVERLAP; Manders’ overlap; MOC) (A’ and B’). (C-E) Graphical summary of whole cell (C) and regional FGFR::VENUS/ CLIP::RAB11 colocalization (D-E; Manders’ overlap) in founder cells treated as indicated. (F-H) Graphical summary of whole cell (F) and regional FGFR::VENUS/ CLIP::RAB4 colocalization (G-H; Manders’ overlap) in founder cells treated as indicated. Data were obtained from 2 independent trials, n > 14. Scale bars are indicated in micrometers. Significance indicated by p-value or a change in lettering (a versus b). Lack of significance indicated by n.s. Significance was determined using one-way ANOVA followed by Tukey multiple comparison test. Numerical values for all graphs can be found in S8 Data. CDK1, Cyclin-dependent Kinase 1; FGF, Fibroblast Growth Factor; n.s., not significant. (PDF) [file pbio.3001029.s003.pdf]

S3 Fig

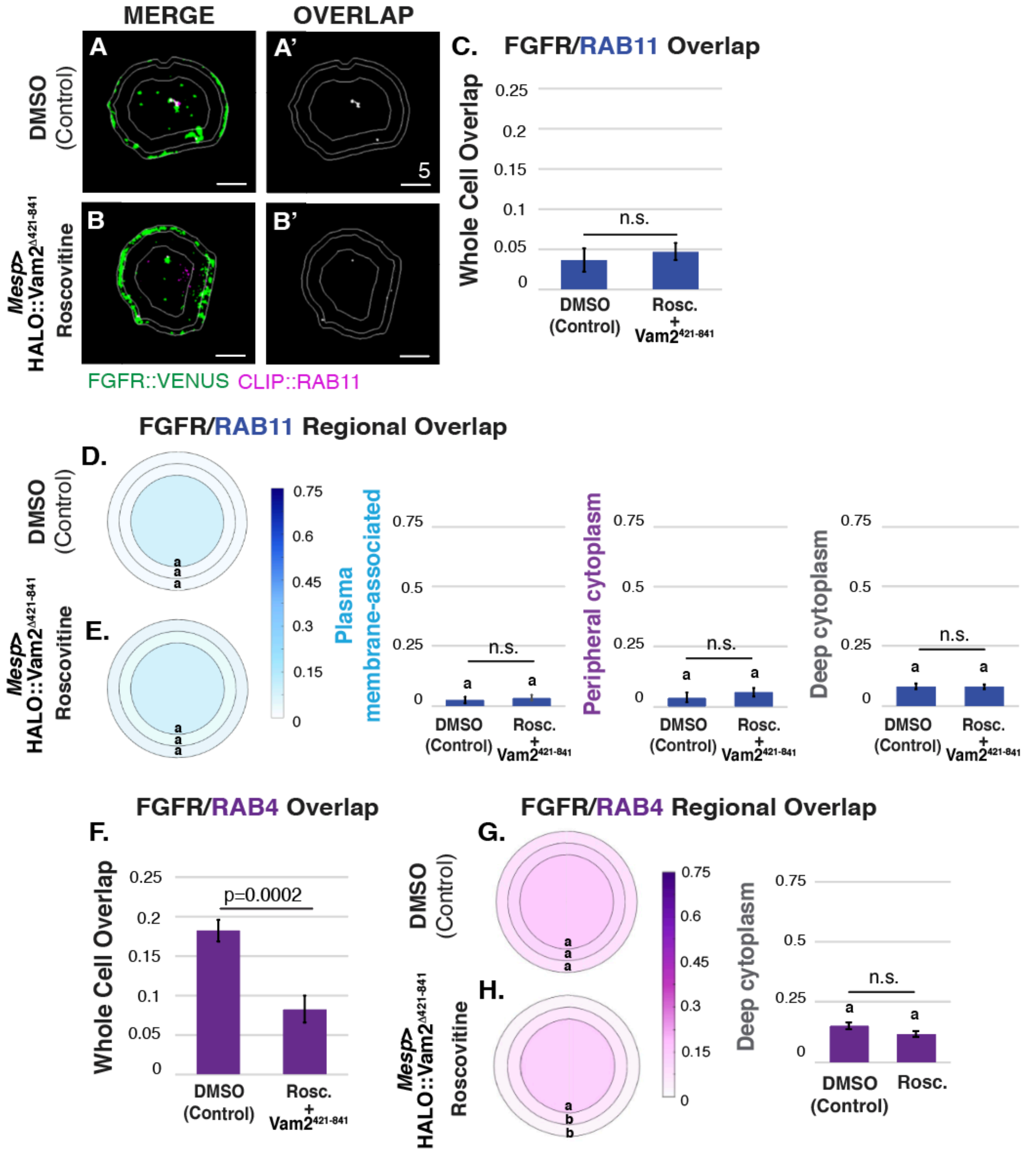

S3 Fig. Inhibition of CDK1 does not impact endosomal maturation or slow recycling of FGF receptors during mitotic entry (Related to Figures 2 & 3). (**A-B'**) Masked/thresholded transverse sections of founder cells electroporated with *Mesp>FGFR::Venus* and *Mesp>HALO::RAB11* and treated as indicated. For clarity, panels showing only co-localized FGFR::VENUS/CLIP::RAB11 puncta are provided (OVERLAP; Manders' overlap; MOC) (**A'&B'**). (**C-E**) Graphical summary of whole cell (**C**) and regional FGFR::VENUS/ CLIP::RAB11 colocalization (**D-E**; Manders' overlap) in founder cells treated as indicated. (**F-H**) Graphical summary of whole cell (**F**) and regional FGFR::VENUS/ CLIP::RAB4 colocalization (**G-H**; Manders' overlap) in founder cells treated as indicated. Data were obtained from 2 independent trials, n>14. Scale bars in micrometers. Significance indicated by p value, or a change in lettering (a vs. b). Lack of significance indicated by n.s. Significance was determined using one-way ANOVA followed by Tukey's multiple comparison test. Numerical values for all graphs can be found in S8 Data.
