# Supplementary material for: Cyclin-dependent Kinase 1 and Aurora Kinase choreograph mitotic storage and redistribution of a growth factor receptor
Source: PLoS Biol. 2021 Jan 4;19(1):e3001029. doi: 10.1371/journal.pbio.3001029 (PMC7808676; doi:10.1371/journal.pbio.3001029)

S4 Fig. Prolongation of CDK1 activity leads to excessive FGFR internalization and blocks TVC induction (Related to Figure 2). (**A-B'**) Ventral projections and lateral sections for founder cells electroporated as indicated. Dashed lines (**A&B**; orange) indicate position of sections (**A'&B'**). (**C**) Graphical summary of regional FGFR::VENUS enrichment for founder cells electroporated as indicated (deep cytoplasm; p=0.264). Data were obtained from 2 independent trials, n>7. (**D**) Graphical summary of mitotic arrest observed for founder cells electroporated. Data were obtained from 3 independent trials, n>22 per trial. (**E-F''**) Representative micrographs of late tailbud embryos showing cranial-cardiac progenitor induction (indicated by overlap of *Mesp>GFP* and *FoxF>RFP* reporter expression along with migration into the head/trunk region) versus non-induced pre-cardiac founder lineage cells (indicated by *Mesp*>GFP reporter expression alone along with lack of migration) in embryos co-electroporated with either *Mesp>LacZ* or *Mesp>CyclinB*$^{\Delta 90}$ as indicated [20,40,41,21]. ATM=Anterior Tail Muscle Cell, TVC = cranial cardiac progenitor. Note that prolongation of CDK1 activity appears to disrupt induction. This may be due to failure of transgenic cells to properly exit mitosis or it may reflect observed FGFR internalization. (**G-H**) Graphical summary of mitotic arrest and heart progenitor induction in embryos co-transfected as indicated. Data were obtained from 3 independent trials, n>8 per trial. Arrested *Mesp>CyclinB*$^{\Delta 90}$ transgenic embryos (**A-C**) were fixed and analyzed at Hotta Stage 16 [22], ~1 hour after control cells (*Mesp>LacZ*) complete asymmetric division. Scale bars are indicated in micrometers. Significance was determined using one-way ANOVA followed by Tukey's multiple comparison test. Numerical values for all graphs can be found in S9 Data. Error bars represent S.E.M.

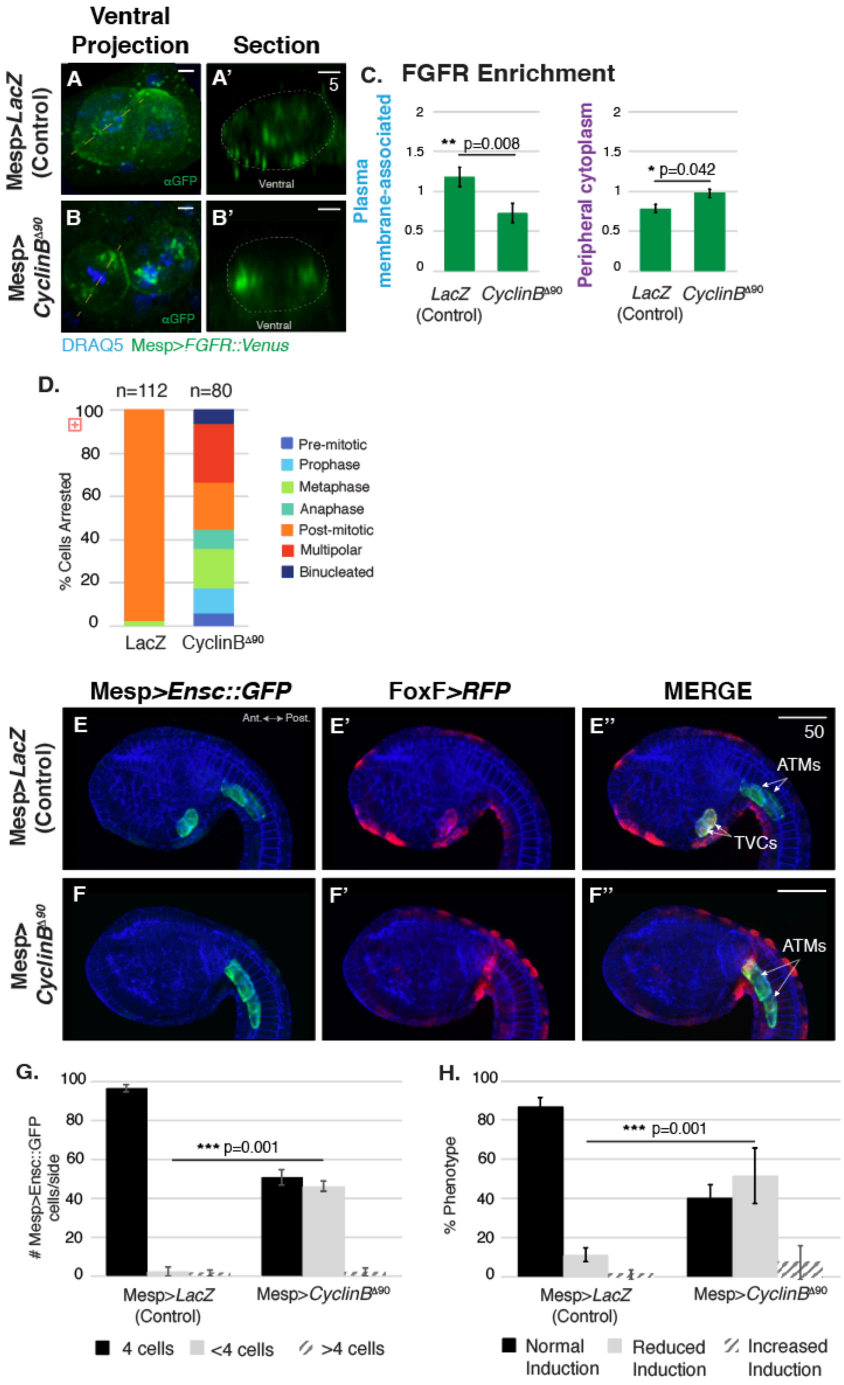

Supplement: S4 Fig — (A-B’) Ventral projections and lateral sections for founder cells electroporated as indicated. Dashed lines (A and B; orange) indicate position of sections (A’ and B’). (C) Graphical summary of regional FGFR::VENUS enrichment for founder cells electroporated as indicated (deep cytoplasm; p = 0.264). Data were obtained from 2 independent trials, n > 7. (D) Graphical summary of mitotic arrest observed for founder cells electroporated. Data were obtained from 3 independent trials, n > 22 per trial. (E-F”) Representative micrographs of late tailbud embryos showing cranial-cardiac progenitor induction (indicated by overlap of Mesp> Ensc::GFP and FoxF>RFP reporter expression along with migration into the head/trunk region) versus noninduced precardiac founder lineage cells (indicated by Mesp>Ensc::GFP reporter expression alone along with lack of migration) in embryos coelectroporated with either Mesp>LacZ or Mesp>CyclinBΔ90 as indicated [20,40,41,21]. Note that prolongation of CDK1 activity appears to disrupt induction. This may be due to failure of transgenic cells to properly exit mitosis or it may reflect observed FGFR internalization. (G-H) Graphical summary of mitotic arrest and heart progenitor induction in embryos cotransfected as indicated. Data were obtained from 3 independent trials, n > 8 per trial. Arrested Mesp>CyclinBΔ90 transgenic embryos (A-C) were fixed and analyzed at Hotta Stage 16 [22], approximately 1 hour after control cells (Mesp>LacZ) complete asymmetric division. Scale bars are indicated in micrometers. Significance was determined using one-way ANOVA followed by Tukey multiple comparison test. Numerical values for all graphs can be found in S9 Data. Error bars represent SEM. ATM, Anterior Tail Muscle Cell; CDK1, Cyclin-dependent Kinase 1; FGFR, Fibroblast Growth Factor Receptor; SEM, standard error of mean; TVC, Trunk ventral cell/Cranial-cardiac progenitor. (PDF) [file pbio.3001029.s004.pdf]
