# Supplementary material for: Cyclin-dependent Kinase 1 and Aurora Kinase choreograph mitotic storage and redistribution of a growth factor receptor
Source: PLoS Biol. 2021 Jan 4;19(1):e3001029. doi: 10.1371/journal.pbio.3001029 (PMC7808676; doi:10.1371/journal.pbio.3001029)
Supplement: S5 Fig — (A-B’) Lateral sections and graphical summary of regional FGFR::VENUS enrichment for founder cells electroporated with Mesp>FGFR::Venus alone or in combination with Mesp>HALO::Vam2421-841 and treated with vehicle (DMSO) or Roscovitine (14 μmol/L) as indicated. Mesp>HALO::Vam2421-841 alone also resulted in a modest, but not significant, increase in plasma membrane-associated FGFR::VENUS. Because phalloidin staining obscures FGFR::VENUS localization, red dashed lines were used to indicate phalloidin-stained cell membranes (A-B). Some regions are labeled with an a or b to denote significant changes (p < 0.05) that occurred within this region across stages. Other regions are labeled n.s. to denote that no significant changes occurred for the indicated stages. Significance was determined using one-way ANOVA followed by Tukey multiple comparison test. (C) Quantification of the FGFR::VENUS enrichment in the plasma membrane-associated region of founder cells electroporated and treated as indicated. Significance was determined using one-way ANOVA followed by Tukey multiple comparison test. Numerical values for all graphs can be found in S10 Data. Scale bars are indicated in micrometers. CDK1, Cyclin-dependent Kinase 1; FGF, Fibroblast Growth Factor. (PDF) [file pbio.3001029.s005.pdf]

S5 Fig

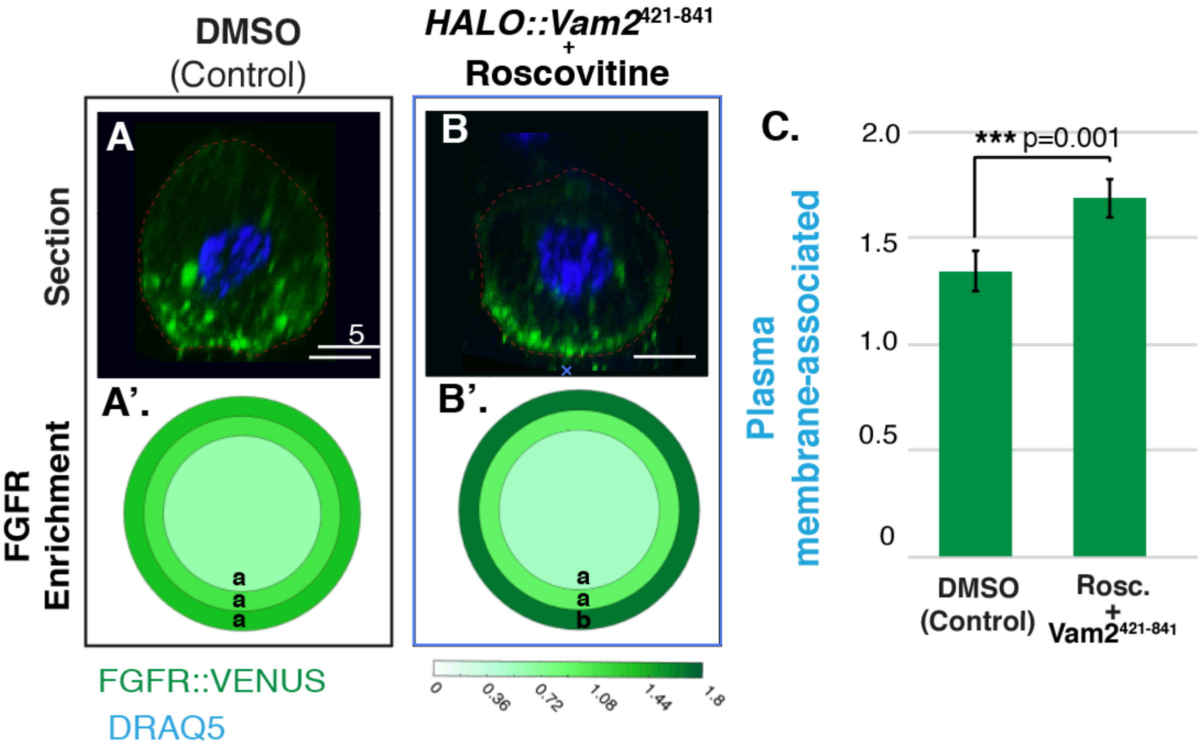

S5 Fig. Inhibition of both CDK1 Kinase activity and lysosomal degradation increases the plasma membrane-associated enrichment of FGF receptors. (**A-B'**) Lateral sections and graphical summary of regional FGFR::VENUS enrichment for founder cells electroporated with *Mesp>FGFR::Venus* alone or in combination with *Mesp>HALO::Vam2*$^{421-841}$ and treated with vehicle (DMSO) or roscovitine (14μmol/L) as indicated. *Mesp>HALO::Vam2*$^{421-841}$ alone also resulted in a modest, but not significant, increase in plasma membrane-associated FGFR::VENUS. Because phalloidin staining obscures FGFR::VENUS localization, red dashed lines were used to indicate phalloidin stained cell membranes (**A-B**). Some regions are labeled with an a or b to denote significant changes (p<0.05) that occurred within this region across stages. Other regions are labeled n.s. to denote that no significant changes occurred for the indicated stages. Significance was determined using one-way ANOVA followed by Tukey's multiple comparison test. (**C**) Quantification of the FGFR::VENUS enrichment in the plasma membrane-associated region of founder cells electroporated and treated as indicated. Significance was determined using one-way ANOVA followed by Tukey's multiple comparison test. Numerical values for all graphs can be found in S10 Data. Scale bars are indicated in micrometers.
