# Supplementary material for: Cyclin-dependent Kinase 1 and Aurora Kinase choreograph mitotic storage and redistribution of a growth factor receptor
Source: PLoS Biol. 2021 Jan 4;19(1):e3001029. doi: 10.1371/journal.pbio.3001029 (PMC7808676; doi:10.1371/journal.pbio.3001029)
Supplement: S6 Fig — (A-D”) Representative micrographs of late tailbud embryos showing induced cranial-cardiac progenitors (TVCs, arrowheads point to cells showing overlapping Mesp>GFP and FoxF>RFP reporter expression) versus noninduced anterior muscle lineage cells (ATMs, arrows point to cells showing Mesp>GFP reporter expression alone) in embryos coelectroporated with Mesp>LacZ (n = 258), HALO::Rab4 (n = 235), HALO::Rab4S199A/T200A (n = 277), or HALO::Rab4S199D/T200D (n = 130) as indicated [20,40,41,21]. (E) Graphical summary of heart progenitor induction in embryos cotransfected as indicated. Embryos electroporated with HALO::Rab4S199A/T200A show increased induction as indicated by the increased proportion of cells with overlapping Mesp>Ensc::GFP and FoxF>RFP in comparison to control embryos electroporated with Mesp>LacZ (p = 0.02) or HALO::Rab4 (p = 0.02). Embryos electroporated with HALO::Rab4S199D/T200D show decreased induction as indicated by the increased proportion of cells with Mesp>Ensc::GFP but no FoxF>RFP in comparison to control embryos electroporated with Mesp>LacZ (p = 0.0001) or HALO::Rab4 (p = 0.006). Data were obtained from 3 independent trials, n > 31 per trial. Scale bars are indicated in micrometers. Significance was determined using a t test with an arcsine square root transformation. Numerical values for all graphs can be found in S11 Data. Error bars represent SEM. ATM, Anterior Tail Muscle Cell; SEM, standard error of mean; TVC, Trunk ventral cell/Cranial-cardiac progenitor. (PDF) [file pbio.3001029.s006.pdf]

S6 Fig

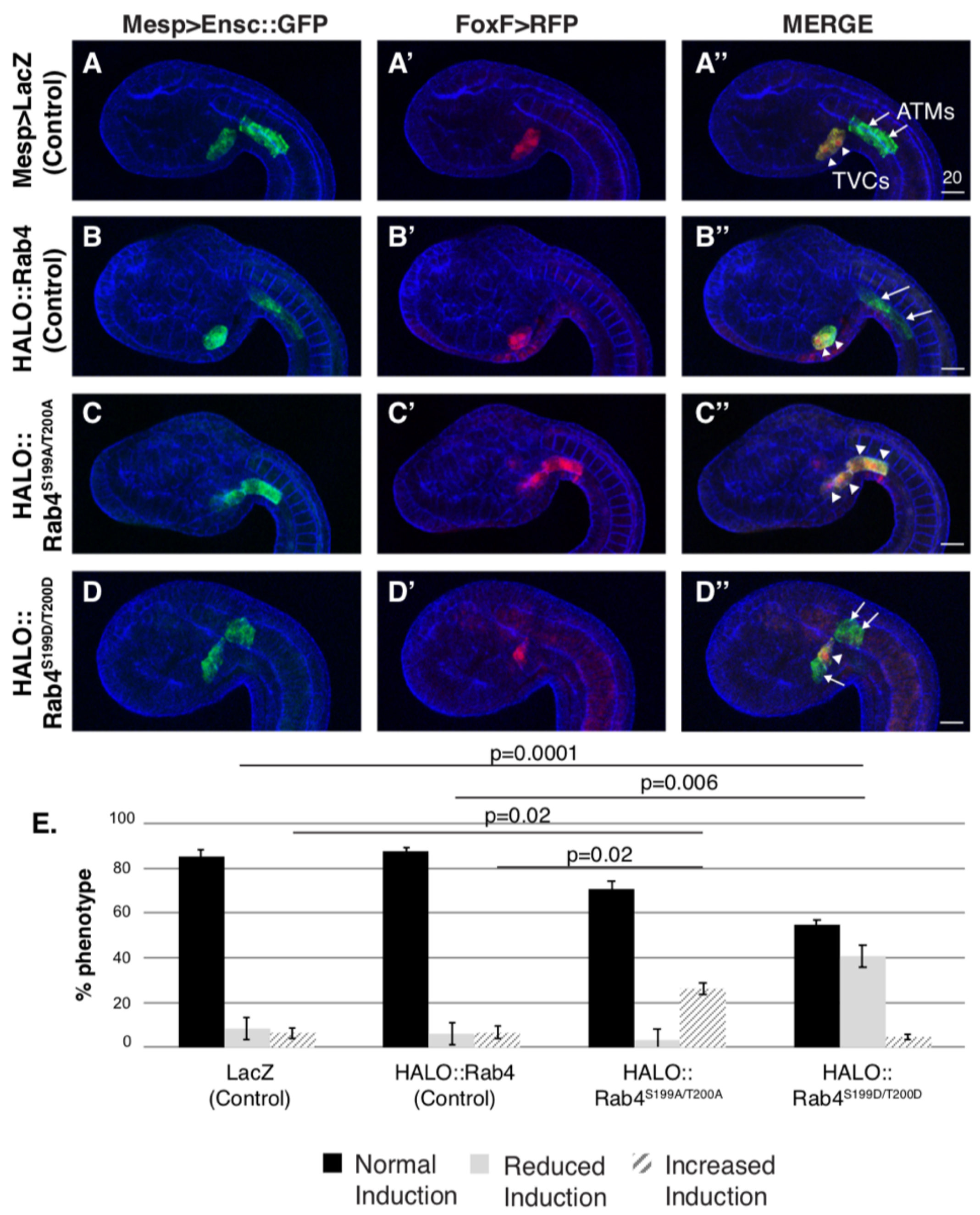

S6 Fig. RAB4 phosphomutants impact TVC induction (Related to Figure 4). (**A-D''**) Representative micrographs of late tailbud embryos showing induced cranial-cardiac progenitors (TVCs, arrowheads point to cells showing overlap of *Mesp>GFP* and *FoxF>RFP* reporter expression) versus non-induced anterior muscle lineage cells (ATMs, arrows point to cells showing *Mesp*>GFP reporter expression alone) in embryos co-electroporated with Mesp>*LacZ* (n = 258), *HALO::Rab4* (n = 235), *HALO::Rab4*$^{S199A/T200A}$ (n = 277), or *HALO::Rab4*$^{S199D/T200D}$ (n = 130) indicated [20,40,41,21]. (**E**) Graphical summary of heart progenitor induction. Embryos electroporated with *HALO::Rab4*$^{S199A/T200A}$ show increased induction as indicated by the increased proportion of cells with overlapping Mesp>*Ensc::GFP* and FoxF>*RFP* in comparison to control embryos electroporated with Mesp>*LacZ* or *HALO::Rab4.* Embryos electroporated

with *HALO::Rab4*$^{S199D/T200D}$ show decreased induction as indicated by the increased proportion of cells with Mesp>*Ensc::GFP* but no FoxF>*RFP* in comparison to control embryos electroporated with Mesp>*LacZ* or *HALO::Rab4*. Data were obtained from 3 independent trials, n>31 per trial. Scale bars are indicated in micrometers. Significance was determined using a t-test with an arcsine square root transformation. Numerical values for all graphs can be found in S11 Data. Error bars represent S.E.M.
