# Supplementary material for: Cyclin-dependent Kinase 1 and Aurora Kinase choreograph mitotic storage and redistribution of a growth factor receptor
Source: PLoS Biol. 2021 Jan 4;19(1):e3001029. doi: 10.1371/journal.pbio.3001029 (PMC7808676; doi:10.1371/journal.pbio.3001029)
Supplement: S7 Fig — (A-C) Graphical summary and quantitative analysis of regional FGFR::VENUS enrichment for founder cells electroporated with Mesp>FGFR::Venus and treated with vehicle (DMSO) or AMG-900 (10 μmol/L) as indicated. (D) Quantification of regional FGFR::VENUS/CLIP::RAB11 overlap in founder cells electroporated and treated as indicated. (E-G) Masked/thresholded transverse sections and quantification of regional FGFR::VENUS/CLIP::RAB4 overlap for founder cells electroporated and treated as indicated. MOCs for whole cell analysis are indicated (E and F) Note that treatment with VX-680 had no significant impact on Rab4 colocalization (E-G). Treatment with AMG-900 also had no significant impact [whole cell overlap for DMSO-treated cells MOC = 0.155 ± 0.019 (n = 7) and AMG-900 treated cells MOC = 0.112 ± 0.015 (n = 3) p = 0.118]. (H-K) Graphical summary and quantification of regional FGFR::VENUS/CLIP::RAB7 overlap in founder cells electroporated and treated as indicated. (L-O) Graphical summary and quantification of regional FGFR::VENUS/CLIP::RAB7 overlap in founder cells electroporated and treated as indicated. Data were obtained from 2 independent trials. n = number of founder cells analyzed. Scale bars are indicated in micrometers. Significance indicated by asterisk and/or change in letter. n.s., not significant. Significance was determined using one-way ANOVA followed by Tukey multiple comparison test. Numerical values for all graphs can be found in S12 Data. FGF, Fibroblast Growth Factor; MOC, Manders’ overlap coefficient. (PDF) [file pbio.3001029.s007.pdf]

S7 Fig

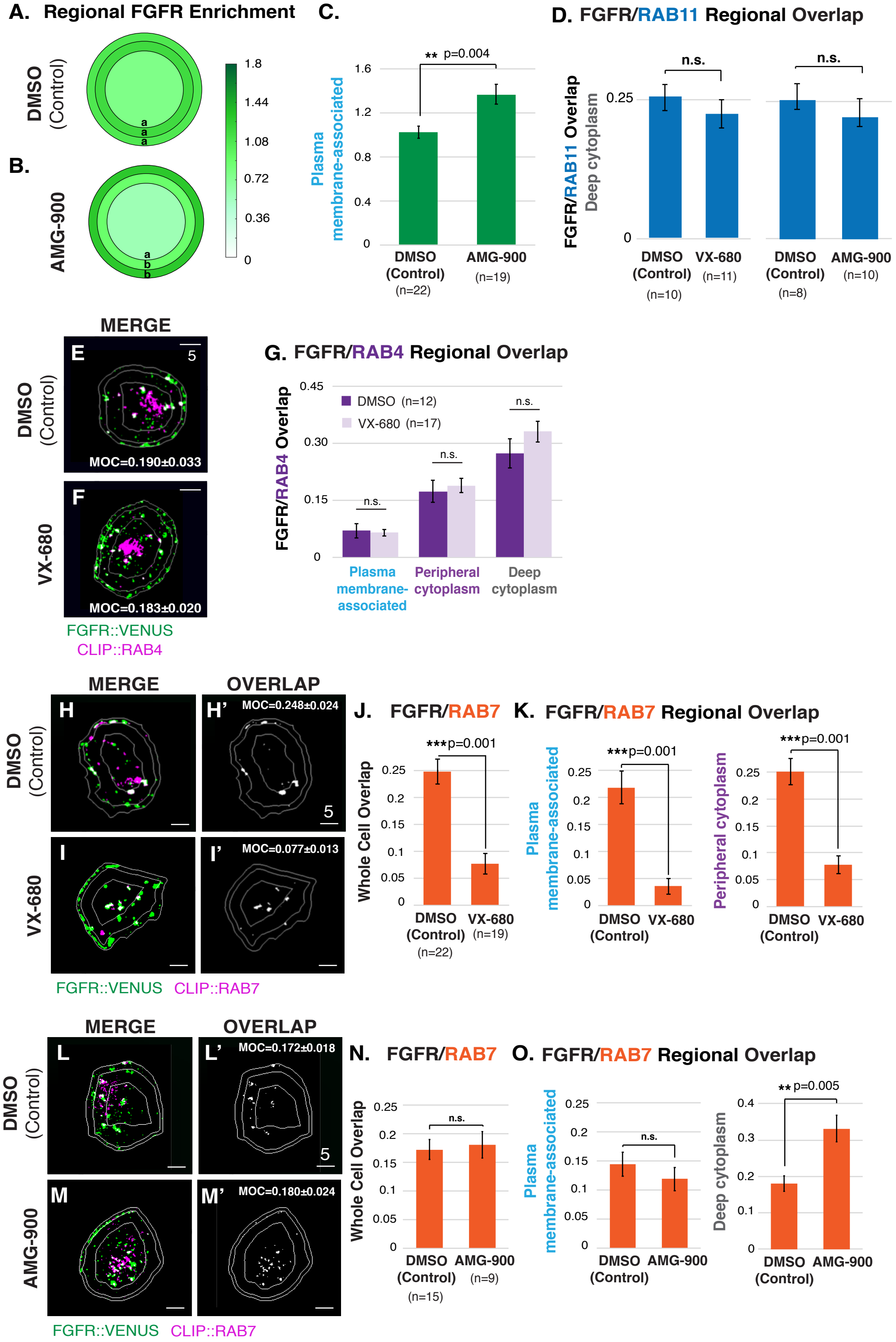

S7 Fig. Inhibition of Aurora Kinase activity does not impact fast recycling of FGF receptors during mitotic entry or RAB7 or RAB11 overlap in the deep cytoplasm (Related to Figure 4). (A-C) Graphical summary and quantitative analysis of regional FGFR::VENUS enrichment for founder cells electroporated with *Mesp>FGFR::Venus* and treated with vehicle (DMSO) or AMG-900 (10 μmol/L) as

indicated. (**D**) Quantification of regional FGFR::VENUS/CLIP::RAB11 overlap in founder cells electroporated and treated as indicated. (**E-G**) Masked/thresholded transverse sections and quantification of regional FGFR::VENUS/CLIP::RAB4 overlap for founder cells electroporated and treated as indicated. Manders' overlap coefficient's (MOCs) for whole cell analysis are indicated (**E & F**) Note that treatment with VX-680 had no significant impact on Rab4 colocalization (**E-G**). Treatment with AMG-900 also had no significant impact [whole cell overlap for DMSO-treated cells MOC=0.155± 0.019 (n=7) and AMG-900 treated cells MOC=0.112 ± 0.015 (n=3) p=0.118]. (**H-K**) Graphical summary and quantification of regional FGFR::VENUS/CLIP::RAB7 overlap in founder cells electroporated and treated as indicated. (**L-O**) Graphical summary and quantification of regional FGFR::VENUS/CLIP::RAB7 overlap in founder cells electroporated and treated as indicated. Data were obtained from 2 independent trials. n= number of founder cells analyzed. Scale bars are indicated in micrometers. Significance indicated by asterisks, n.s.= not significant. Significance was determined using one-way ANOVA followed by Tukey's multiple comparison test. Numerical values for all graphs can be found in S12 Data.
