# Supplementary material for: Cyclin-dependent Kinase 1 and Aurora Kinase choreograph mitotic storage and redistribution of a growth factor receptor
Source: PLoS Biol. 2021 Jan 4;19(1):e3001029. doi: 10.1371/journal.pbio.3001029 (PMC7808676; doi:10.1371/journal.pbio.3001029)
Supplement: S1 Table — (PDF) [file pbio.3001029.s008.pdf]

S1 Table. Key Reagents/Resources used in this study.

REAGENT or RESOURCE	SOURCE	IDENTIFIER
Antibodies		
GFP Tag Monoclonal Antibody (3E6)	Molecular Probes	Cat#: A-11120<br>RRID:AB_221568
Donkey anti-Mouse IgG (H+L) Highly Cross-Adsorbed Secondary Antibody, Alexa Fluor 488	Molecular Probes	Cat#: A-21202<br>RRID:AB_141607
Chemicals, Peptides, and Recombinant Proteins		
Roscovitine	Sigma-Aldrich	Cat#: R7772
VX-680 (Tozasertib)	Selleckchem.com	Cat#: S1048
AMG-900	Selleckchem.com	Cat#:S2719
DMSO	Sigma-Aldrich	Cat#:D2650
CLIP-Cell TMR-Star	New England Biolabs	Cat#: S9219S
Brefeldin A	Sigma-Aldrich	Cat#: B7651
AlexaFluor Phalloidin 633	Molecular Probes	Cat#: A22284
DRAQ5	Cell Signaling Technologies	Cat#: 4084
Experimental Models: Organisms/Strains		
C. robusta	M-Rep	N/A
Oligonucleotides		
Rab11_BamHI_F: 5'-ATTGGGATCCAATGGGAACCAAAGATGACG-'3	Sigma-Aldrich	N/A
Rab11_BlpI_R: 5'-GAAGCTCAGCTAGATATTTTTACAACATTG-3'	Sigma-Aldrich	N/A
Rab7_BamHI_F: 5'-AAAGGATCCAATGGCGTCAAGGAA-3'	Sigma-Aldrich	N/A
Rab7_BlpI_R: 5'-TTTGGATCCAGCATGAGCAATCTC-3'	Sigma-Aldrich	N/A
Rab7noBlp_F: 5'-TATCCCATACTTTGAATGTTCAGCGAAGGACAACATC-3'	Integrated DNA Technologies, Inc.	N/A
Rab7noBlp_R: 5'-GATGTTGTCCTTCGCTGAACATTCAAAGTATGGGATA-3'	Integrated DNA Technologies, Inc.	N/A
Rab4_BamHI_F: 5'-AAAGGATCCTATGAGTGAAAGATACGA-3'	Sigma-Aldrich	N/A
F ClipSnapNot: 5'-AAAGCGGCCGCCCAAACCATGGACAAAGACTGCGAAATG-3'	Integrated DNA Technologies, Inc.	N/A
R HaloCLIP Bam: 5'–AAAGGATCCAGGATAACTCGAGATCTGAGTCCG-3'	Integrated DNA Technologies, Inc.	N/A
Rab4_BlpI_R: 5'-TTTGCTCAGCTCAAGCACATTTACATT-3'	Sigma-Aldrich	N/A
Rab4ST_AA_F:5'-GCATTAAGACAATTACGCGCCGCAAATCGACAAAGGAACGTC-3'	Integrated DNA Technologies, Inc.	N/A
Rab4ST_AA_R: 5'-AAAGCTCAGCAAAGATTGACCAATCAGGAGTGTCG-3'	Integrated DNA Technologies, Inc.	N/A

Rab4S_A_F: 5'-GCATTAAGACAATTACGCGCCACAAATCGACAAAGGAAC-3'	Integrated DNA Technologies, Inc.	N/A
Rab4S_A_R: 5'-GTTCCTTTGTCGATTTGTGGCGCGTAATTGTCTTAATGC-3'	Integrated DNA Technologies, Inc.	N/A
Rab4ST_DD_F: 5'-GCATTAAGACAATTACGCGACGACAATCGACAAAGGAACGTC-3'	Integrated DNA Technologies, Inc.	N/A
Rab4ST_DD_R: 5'-GACGTTCCTTTGTCGATTGTCGTCGCGTAATTGTCTTAATGC-3'	Integrated DNA Technologies, Inc.	N/A
VAM2_Forward: 5'-AAAGGATCCGAATAATATGAACCACTGGGAGG-3'	Integrated DNA Technologies, Inc.	N/A
VAM2_Reverse: 5'-AAAGCTCAGCAAAGATTGACCAATCAGGAGTGTCG-3'	Integrated DNA Technologies, Inc.	N/A
CKI_NotI_F: 5'-AAAGCGGCCGCAATGGTTCCCCCACCTTCGTACAATGC-3'	Integrated DNA Technologies, Inc.	N/A
CKI_Blp_R: 5'-AAAGCTCAGCTTCAATGAGTTGGAAGCGACCATGG-3'	Integrated DNA Technologies, Inc.	N/A
CyBDN Not1F: 5'-AAAGCGGCCGCAAAGATGCAACTCCAGAAACCAACAACCACC-3'	Integrated DNA Technologies, Inc.	N/A
CyBDN EcoR1R: 5'-AAAGAATTCATAACTTGTGACTTGCTGCCTGCTCTGC-3'	Integrated DNA Technologies, Inc.	N/A
Recombinant DNA		
Unigene collection clone (Rab4)	Cogenics	VES67_I21
Unigene collection clone (Rab7)	Cogenics	VES63_C01
Unigene collection clone (Rab11)	Cogenics	VES68_O04
Unigene collection clone (Vam2)	Cogenics	VES88_L15
CLIP-rGBD Rho	W. M. Bement	N/A
HALO-rGBD	W. M. Bement	N/A
Mesp>LacZ	(Davidson et al., 2005)	N/A
Mesp>FGFR::Venus	(Cota and Davidson, 2015)	N/A
Mesp>CLIP::Rab4	This study	N/A
Mesp>HALO::Rab4	This study	N/A
Mesp>HALO::Rab4$^{S199A/T200A}$	This study	N/A
Mesp>HALO::Rab4^{S199A}	This study	N/A
Mesp>HALO::Rab4$^{S199D/T200D}$	This study	N/A
Mesp>E-Cadherin::GFP	(Norton et al., 2013)	N/A
Mesp>CLIP::Rab7	This study	N/A
Mesp>CLIP::Rab11	This study	N/A
Mesp>HALO::Vam2$^{421\text{-}841}$	This study	N/A
Mesp>Cdki(p27)	This study	N/A
Mesp>CyclinB$^{\Delta 90}$	This study	N/A
Software and Algorithms		

FIJI(ImageJ)	NIH	RRID:SCR_002285
Matlab	Mathworks	RRID:SCR_001622
Other		
35mm glass-bottom culture dish	MatTek	Cat#: P35G-0-10-C
