# Supplementary material for: Cyclin-dependent Kinase 1 and Aurora Kinase choreograph mitotic storage and redistribution of a growth factor receptor
Source: PLoS Biol. 2021 Jan 4;19(1):e3001029. doi: 10.1371/journal.pbio.3001029 (PMC7808676; doi:10.1371/journal.pbio.3001029)
Supplement: Attachment — Submitted filename: Response to reviewers PLOS CDC 929.docx [file pbio.3001029.s021.docx]

Di Jiang, Ph.D

*PLOS Biology*

1160 Battery Street
Koshland Building East, Suite 225
San Francisco, CA 94111
[plosbiology@plos.org](mailto:plosbiology@plos.org)

Dear Dr. Jiang-

We would like to thank you and the other editors at *PLOS Biology* for your thoughtful comments on our manuscript, "Mitotic Kinases choreograph receptor storage and redistribution" (PBIOLOGY-D-20-00218R1).

We were very pleased to see the strong support for our paper from all four of our reviewers and their recognition that “this manuscript is potentially of great interest to cell and developmental biology.” We thank you for the opportunity to address the concerns they have raised.

The primary concern raised by the reviewers pertained to our use of a single inhibitor, VX-680, to investigate the role of Aurora Kinase in regulating FGFR trafficking during founder cell division. To address this key concern, we have repeated several experiments using an alternative Aurora Kinase inhibitor, AMG-900. We have also made substantial edits to improve the clarity of the manuscript as suggested by the reviewers.

Per your request, we have attached a point-by-point response to this letter thoroughly addressing reviewers’ comments.

Once again, we would like to thank you for your consideration of this manuscript for publication in *PLOS Biology* as well as your patience throughout this process as our revisions were delayed as a result of institutional restrictions put in place in response to the COVID-19 pandemic.

Sincerely,

*Christina Cota*	Corresponding Author:

Asst. Prof. Dept. of Biology	*Brad Davidson*

Colby College	Assoc. Prof. Dept. of Biology

5727 Mayflower Hill	Swarthmore College

Waterville, ME. 04901	500 College Ave.

Email: [cdcota@colby.edu](mailto:cdcota@colby.edu)	Swarthmore PA. 19081

Office: 207-859-5727	Email: [bdavids1@swarthmore.edu](mailto:bdavids1@swarthmore.edu)

Cell:  607-262-6136	Office: 610-690-5718

Cell:  520-300-1610

Response to Reviewers

Reviewer #1: As someone who has worked in mitosis, polarity and membrane trafficking, this manuscript is potentially of great interest to cell and developmental biology.  However, the major weakness is that the manuscript is incredibly hard to understand and the results are not always clearly presented.  So, I fear that the non-specialist will not be able to follow it without substantial improvements to the clarity of presentation.  

Comments:

1.  To make their findings more understandable to the non-specialist, the authors could consider converting part of the current Figure S1 into a revised Fig 1, to show where these founder cells are located within the embryo, and to show the cross-section of the wild type FGFR::Venus at interphase, metaphase and telophase, which is currently only schematized in Fig 1A.  The authors should not assume that the reader has read and can recall their previous papers.  Rather, they should feel comfortable in simply showing new versions of previously demonstrated data or schematics again, at least of the wild-type cells and what happens to them during mitosis, so that the basic phenomenon is clearly established in Fig 1 without needing to read the text or previous papers.  For example, why does the previous Dev Cell paper show a schematic of a the Ciona embryo founder cells and their location within the embryo in Fig 1A, while this new paper does not?  It would also be worth showing
new examples of the Fig 1B-D in the Dev Cell paper in this new paper.  In particular, there is no interphase control cell shown for Fig 1B.

While we appreciate the Reviewer’s suggestions for offering additional introductory figures we respectfully disagree. The main reason for this is that unlike our previous papers, the current manuscript is focused on delineating the cell biology of mitotic trafficking, rather than heart progenitor induction. Thus, while our previous papers included many experiments in which we tested for changes in heart progenitor induction and associated changes in heart founder lineage cell migration, these are not relevant to the hypotheses we are testing in the current paper. Because of this shift in focus, we do not think it is particularly pertinent to show the position of heart founder lineage cells in the embryo. We also are avoiding showing more of the 2D sections (including interphase) because these individual sections do not provide an accurate representation of the volumetric data that we quantitated (which is accurately represented in panels C and D). Moreover, we think the schematized versions of founder cell division are easier to follow then using confocal images to illustrate this process**.** Additionally, since Figure 1 already has many images and panels, we are concerned that adding additional panels would make each panel smaller and harder to see. **We have added the following language to the results section to clarify these points.**

“These assays were conducted using *Mesp>FGFR::Venus* transgenic embryos (

Cota and Davidson, 2015; Davidson et al., 2005). Because the Mesp enhancer specifically drives transgene expression in the heart founder cell lineage, we are able to analyze FGFR::VENUS distribution in vivo. Thus, transverse sections (Figure 1B) represent confocal stacks of mitotic founder cells within intact embryos (as illustrated in Figure 1A).”

“Volumetric analysis provided a rigorous and highly reproducible measurement of FGFR::VENUS distribution at each cell cycle stage that is not well-represented by individual transverse sections. Thus, in this and subsequent figures we focus on providing a complete set of graphical data (Figure 1C and D) rather than representative confocal sections for each stage (Figure 1B).”

2.  In general, all of the microscopy images of the founder cells are too small to easily see what is going on.  It would be best to increase the size of each of the panels by around 400% at the expense of the white space currently occupying most of the figures.  Otherwise, it is hard to see any substantive differences between any of the panels, or to see any co-localisation.

Thank you for this suggestion, we have increased the size of these images.

3.  In the previous Dev Cell paper, the FGFR appears to move completely (100%) from the lateral membranes at interphase to endosomes in mitosis (and is asymmetrically distributed).  In this paper, the authors quantify this change but only observe a 30% reduction in plasma membrane-associated FGFR (Fig 1D).  What is the reason for this difference?

This is a good question. As discussed in our Dev Cell paper, FGFR::VENUS appears to be preferentially maintained along the ventral membrane of founder cells (you can see a similar phenotype in Figure 4F) and so it only appears to be internalized along the lateral membranes during mitosis. Since we did not rigorously quantify this shift in our previous study, our description was a qualitative evaluation based on comparing individual orthogonal slices from staged founder cells. Now that we can quantify the change volumetrically, it has become apparent that this shift only represents a ~30% reduction in plasma membrane enrichment. This data may indicate that movement of FGF receptors within the membrane contributes to shifts in FGFR distribution but this is highly speculative. We are currently working on developing methods that would allow us to better assess these finer scale movements in living cells. Until recently, we were unable to conduct live cell analysis because FGFR::VENUS expression in founder cells is very low and antibody staining is required to visualize the tagged proteins. However, now that we are able to disrupt FGFR degradation we should be able to address these mechanisms in future research.

4.  I find the Roscovitine (CDK1 inh.) experiment deeply confusing.  If mitotic entry is blocked, shouldn't the FGFR stay at the plasma membrane?  The fact that it doesn't (and is degraded) is interpreted as these cells being 'blocked in prophase'.  But what is the evidence for this?  Secondly, if active CDK1 is needed to inhibit degradation of FGFR, then why isn't FGFR degraded during interphase, when CDK1 is not active?  I would suggest repeating the Roscovitine experiment in interphase cells - is the FGFR also degraded there?  The authors must already have done this control.  Also, is it clear that VX680 doesn't also lead to inhibition of CDK1 through loss of the positive feedback loop between AuroraA/B and CDK1 activation?  If so, then perhaps the Roscovitine is causing an artefactual result, which the authors should consider seriously before pressing ahead with publication.

The Reviewer raises a number of important concerns in this paragraph that we address below.

1. We were able to use DRAQ-5 staining to observe chromatin and confirm that arrested cells had begun chromosomal compaction associated with prophase. Additionally, we were able to finesse the timing of roscovitine application to coincide with founder cell entry into mitosis and thus enrich the proportion of cells that were arrested at prophase. **We added the following language to clarify this point.**

“By treating late gastrulae (Hotta Stage 14) with a fast-acting CDK1 inhibitor (roscovitine/seliciclib) we were able to block CDK1 activity in mitotic founder cells. Through DRAQ-5 staining we were able to identify treated founder cells displaying chromatin condensation, indicating that they had been arrested in prophase.”

2. Treatment of founder cells with roscovitine during interphase does not result in a loss of FGFR staining. Although we do not show this data, **we have added the following language regarding these results.**

“We also treated embryos with roscovitine during interphase. As predicted by our model, this treatment had no discernable impact on FGFR:VENUS staining (data not shown).”

3. We are confident that feedback inhibition of CDK1 due to VX-680 treatment (or feedback inhibition of AurK due to roscovitine treatment) did not create artefactual results because treatment with these inhibitors (roscovitine vs. VX- 680) led to highly distinctive, non-overlapping effects on FGF receptor distribution and staining levels as detailed in Figures 3 and 5. For example, if VX-680 inhibited CDK1 then we would have expected to observe reduced FGFR:VENUS staining, instead VX-680 had no discernable impact on FGFR::VENUS staining levels (Figure 3).

Reviewer #2 (Emmanuel Boucrot, signed review): Cota, Davidson and colleagues are reporting that mitotic kinases Cdk1 and Aurora are suppressing endosomal degradation and recycling back to the plasma membrane of Fibroblast Growth Factor Receptors (FGFRs) during asymmetric division.  

The authors followed-up on their seminal 2015 paper in which they reported that FGFR are internalised into endosomes during pre-cardiac founder cell division in the chordate Ciona robusta. This leads to an unequal distribution of FGFRs between the two daughter cells, thereby causing differential cell fate, which is critical for proper development. The study of mitotic FGFR distribution in Ciona founder cells is a powerful model of highly physiological relevance.

In the present study, the authors measured with precision FGFR distribution between daughter cells and localisation inside late, fast- and slow-recycling endosomes (Rab7-, Rab4- and Rab11-positive compartment, respectively). They found that the receptors are stored into Rab11- and Rab7-endosomes during early mitosis and recycled back to the plasma membrane during mitotic exit. They further established that the mitotic kinase CyclinB1/CDK1 blocks the Rab4-dependent fast recycling route whereas Aurora controls the fusion back to the plasma membrane from Rab11-positive compartments. They also establish that both kinases controls late endosome to lysosome conversion, tipping the balance towards the formers and thereby sparing FGFRs from degradation.

The work was carefully planned, executed and analysed with the appropriated controls and statistics to support most of the conclusions. The findings are new and deepen significantly our understanding of membrane trafficking during mitosis and endosome inheritance upon cell division.

The only weak part of the manuscript is the section on Aurora kinase (Figure 5):
1. Unlike for CDK1 where the authors backed up their results with small compound inhibitor with a genetic approach (the CyclinBDelta90 construct), all the conclusions on Aurora are based on the inhibitor VX-680. However, as most small compound inhibitor, VX-680 is not strictly specific and also inhibits Src, Abl, GSK3beta as well as few other kinases (see PMID 16424036). Its effect on GSK3beta is potentially relevant, as explained below.
Thus, it would be prudent to confirm the phenotype using a second, chemically unrelated, inhibitor such as AMG-900 (PMID 20935223) that do not share the same secondary targets (p38alpha, DDR1 and 2 and LTK for AMG-900). Alternatively, if possible in Ciona, a genetic approach (depletion, dominant negative or constitutively active constructs) would alleviate concerns about small compound inhibitors.

This was an excellent suggestion and an important concern. We have addressed it by repeating several key experiments using the AMG-900 Aurora Kinase inhibitor as suggested by the Reviewer (Figure 5 & S6). The two AurK inhibitors had a similar impact on FGFR distribution and FGFR::VENUS/CLIP::Rab11 colocalization, further supporting our hypothesis regarding AurK dependent suppression of slow recycling. However, the two inhibitors differentially impacted FGFR::VENUS/CLIP::Rab7 co-localization, leading us to discard our hypothesis regarding AurK dependent promotion of endosomal maturation. We did not attempt to speculate about the differences in the impact of these inhibitors in this regard, instead we chose to alter our model and conclusions. The removal of this aspect of our model did not substantially change our conclusions regarding synergistic regulation of receptor storage by Cdk1 and AurK during mitotic entry or the implications that arise from this model as detailed in the discussion.

2. Contrasted with CDK1 where the authors could confirm that the phenotype is mediated by the phosphorylation of S199 on Rab4, the lack of molecular insight about how Aurora works makes for a weak ending to the paper.
The VX-680 phenotype (Fig. 5A-C) is consistent with a block of Rab11-positive recycling endosome back to the cell surface. This could be a defect in RE transport within the cytoplasm or an inhibition of RE to plasma membrane (PM) fusion. Both of these steps are controlled by Rab11 through its interaction with the exocyst complex (Exo70 etc.), Arf6 and FIP3, 4 and 5 proteins.  
Of potential relevance, FIP5 was reported to be phosphorylated on T276 by GSK3beta during mitosis (PMID 24591568). Interestingly, the motif LLT276*RS read from right to left (kinases obviously read amino acids in both direction) is SRT*LL which could be an Aurora B consensus site [R/K][S/T][I/L/V]. With the dual inhibition of Aurora and GSK3beta by VX-680, it is pressing to rule out any role for GSK3beta (using the CHIR-99021 or BIO inhibitors or genetic perturbations such as knock-down or S9A- or K85A- GSK3beta constitutively active and kinase dead mutants, respectively).
It is also possible that Aurora kinase acts on the SNAREs, which in the case of recycling endosomes are VAMP2 or 3, SNAP23 and Syntaxin 4. Inhibiting VAMP3 function perturbed TfR-containing recycling endosome fusion back to the cell surface during late mitosis (PMID 17483462) and there are links between Aurora and SNARE function in late cytokinesis (PMID 16213214; 19887622).

I appreciate that a detailed study of how Aurora blocks RE fusion to the PM is beyond the scope of this manuscript but addressing point 1 (required for publication in PLOS Biology in my opinion) and providing some insights into point 2 would greatly benefit to the impact and robustness of the paper.

**We agree that this is an important area for future study and have added some language to our discussion in this regard.**

“For instance, Aurora Kinase may suppress slow recycling through direct phosphorylation of Rab11 or Rab 11 effectors such as FIPs, MyosinVB, the kinesin Kif13A, or the exocyst subunit EXOC6 (Galvez et al., 2012, O’Sullivan and Lindsey, 2020). Alternatively, Aurora Kinase may directly disrupt downstream factors associated with delivery of slow recycling endosomes to the plasma membrane including Arf6 and its effectors (Grant and Donaldson, 2009). It’s also possible that Aurora Kinase suppresses slow recycling through a more indirect mechanism similar to documented cascades involved in Aurora Kinase-dependent regulation of cytokinesis (Gromley et al., 2005, Song et al., 2009).”

Minor comments:
- the paragraph title "Endocytic pathways involved in mitotic redistribution of FGFR" (page 4) is misleading as one would expect a study of the pathways by which the receptor enter cells during mitosis (i.e. clathrin-mediated endocytosis, CLIC/GEEC, FEME, macropinocytosis etc..). As the authors studied the endosomal paths used by the receptor (fast- and slow-recycling or degradation), "Endosomal pathways involved in […]" would be more appropriated.
- Page 6 (top) and page 7 (top)  "roscovatine" should be "roscovitine"

The manuscript has been edited to reflect these corrections.

Reviewer #3: This paper from Cota and colleagues sheds light on the regulation of endosomal trafficking and receptor sorting during asymmetric cell division.

Endosomal trafficking recently emerged as a major regulator of morphogenesis thought its effects on signalling, both for symmetric cell division (to ensure equal transmission of morphogen molecules between daughter cells) and for asymmetric cell division (to ensure asymmetric cell fate). However, while decades of cell biology provided a deep understanding of the regulation of trafficking during interphase in cultured cells, very little is known about the regulation of trafficking during mitosis in tissues. This is mostly due to the fact that tissues are less amenable to quantitative imaging studies than cultured cells, but also due to the fact that until recently, endocytic uptake was thought to be shut down during mitosis. This quantitative study addresses this question directly, in the physiological context of asymmetric cell division in the chordate Ciona robusta.

The authors first show using quantitative 3D imaging that FGFR receptors are internalized during early mitosis, before going back to the plasma membrane at the end of mitosis. They then show that internalized receptors do colocalize with recycling endosomes (Rab4-Rab11) and late endosomes (Rab7). They then use drugs to characterize the effect of mitotic kinases (Aurora A and CDK1) on this FGFR trafficking. They then provide evidences that CDK1 inhibits FGFR degradation in lysosomes and inhibit fast recycling through the Rab4 pathway. Last, they provide evidences that Aurora kinase inhibits Rab11-dependant slow recycling, as well as increase receptor trafficking to late, Rab7 compartments.

I think this is an excellent paper addressing a fundamental question of trafficking in a physiologically relevant context. I particularly appreciated the efforts the authors put in being very quantitative. I found particularly fascinating the finding that CDK1 specifically protects from degradation only a subset of receptors (like FGFR) rather that uniformly inhibiting the degradation pathway.  This has important implications for our molecular understanding of morphogenesis, in particular because tissue-scale variations of receptor degradation in lysosomes is thought to control the establishment of morphogen gradients. While I think the study deserves few controls to ensure the specificity of the treatments used (see below), I think this paper fully deserves publication in PloS biology.  

Specific comments:

Main comment: specificity of the CDK1/Aurora treatment

The first part of the study deals with effects ascribed to the CDK1 kinase using the inhibitor Roscovitine. It is my understanding that this inhibitor is broad and can target other CDK, including CDK2 and CDK5 as well as other kinases (ERK2), albeit with less affinity (Meijer et al, Eur J Biochem. 1997). To address this problem, the authors nicely made use of the CyclinBDelta90 overexpression, which shows increased FGFR signals, in good agreement with their Roscovitine data (Fig 3I). But this panel i) does not have a control (meaning a live FGFR::VENUS expressing cells, showing the near absence of signal) and ii) is not quantified (it should be easy enough to quantify as the authors did in Fig 3E).

We appreciate this concern and need to clarify that under normal wild-type conditions, FGFR::VENUS expression is very low and no expression can be detected without antibody staining. Thus, FGFR::VENUS signal cannot be detected or quantified in live, wild-type cells.

Additionally, control wild-type FGFR::VENUS samples were monitored in parallel to the CyclinBDelta90 samples to ensure that no signal was present as seen previously. **We have modified the language in the results section (as indicated below in red font) to clarify these points.**

“The level of FGFR::VENUS expression in founder cells is very low, precluding live imaging analysis. Instead, transgenic *Mesp>FGFR::Venus* embryos were fixed at 15-min intervals spanning founder cell mitosis and co-stained with an anti-GFP antibody to visualize FGFR:VENUS and a chromatin marker (DRAQ5) to facilitate precise mitotic staging

(Cota and Davidson, 2015; Hotta et al., 2007).”

“Expression of Cyclin B^∆90^ led to a dramatic increase in FGFR::VENUS signal, allowing direct observation of FGFR::VENUS in live, unstained embryos (Figure 3I). As seen previously, no FGFR::VENUS signal was detected in live, matched controls (data not shown).”

I think this part of the work deserves further characterization to ensure that the effects are indeed CDK1-specific. First, by quantifying the CyclinBDelta90 effect, but also by using other CDK1 inhibitors with a different spectrum like BMS-265246 or R547 (there are probably others). Obviously, it is probably not worth redoing ALL the Roscovitine experiments with another inhibitor, but at least Figure3 A,B-D would be nice.

Although we appreciate this concern, we strongly feel that the addition of the CyclinBDelta90 assay provides additional strong support for our hypotheses regarding the proposed role of CDK1 in suppressing lysosomal degradation (as also asserted by the second Reviewer). As we have now clarified in the revised manuscript (see above), the ability to see FGFR::VENUS in live samples is extraordinary and this experiment was controlled.

Similarly, the last figure deals with Aurora inhibition and all data solely comes from one inhibitor, VX-680. This inhibitor inhibits Aurora in the nM range in vitro, which is orders of magnitude below the concentration used by the authors (20uM). This increases the chances of off-targets, which have been reported for this molecule (Cheetham GM, et al. Cancer Lett. 2007)(obviously we do not know if it's true in Ciona as well, perhaps the authors do). I think it would strengthen the paper to confirm these results with either another inhibitor, or a dominant active/inactive Aurora for instance.

This is an excellent point that we have addressed in reference to the same concern raised by Reviewer 2 (see our detailed response above).

Minor comments:
-3D segmentation. I really appreciated the care shown by the authors to use unbiased, user-free thresholding methods. However, I did not understand how the authors segmented the plasma membrane from the VGFR staining (i.e. I don't understand how they come up with the white perimeter of the cell from the green segmented region in Fig 1B'). Please explain in a bit more details.

Although it is not apparent in this image, there was a faint FGFR::VENUS signal associated with the cell as a whole that allowed us to segment it from background. The program used for segmentation was able to detect this difference even though it is difficult to see without added contrast. In addition, cell masks were manually inspected to confirm the accuracy of segmentation for each cell that we analyzed.

-I really like the representation and colour coding scheme chosen by the authors in Fig1C.  I think it would be nice to add a snapshot of a cell for each mitosis phase to appreciate the raw data along the cell cycle (in supplementary material at least).
In addition, I think the "a, b, ns" in Fig 1C and D-D'' (and the rest of the paper) are a bit confusing. Since the authors used different colours when differences are statistically significant with the previous phase, why not simply stating it in the legend? The graph makes the point anyway.

We appreciate these suggestions, however as we stated in response to the first Reviewer, the 2D sections do not accurately capture volumetric data which are precisely represented by our graphical representations. Furthermore, adding these snapshots would make the figures unwieldy and require us to use small panels, reducing overall visibility and clarity.

With respect to the “a, b, ns” notation used in figures to indicate significance, although we agree that the letters might be confusing at first, we think that since we use them consistently they will provide readers with a usable sense of significant differences. The shades of color represent a gradual scale of intensity (shown in each figure) and do not indicate significance. The graphs alone do not give the sense of the spatial nature of the changes.

- The authors are excellent at testing the significance relevance of nearly every observation, but I could not find the number of cells analysed for each condition in Fig 1D-D'', Fig 2 and Fig 4C.

Thank you for this suggestion, the Figure legends have been edited to include these data.

-Fig3K: Why is there DMSO in the control if there is no drug in the Vam2 treatment?

Thank you for this comment, this issue has been corrected in the Figure.

-Fig3 A',B',C',G' it seems that there is some Rab7 signal (magenta) within the nucleus (Cyan). Why is that ?

This is a good question. It is unclear at this point why we see chromatin-associated RAB localization but we can assume that during many of the stages we analyzed, breakdown of the nuclear envelop would permit endosomes to mix in with the chromatin. The localization of RAB proteins in mitotic cells does not follow the patterns established through extensive characterization of endosomal trafficking during interphase. Indeed, RAB proteins, have been implicated in a number of mitotic processes (nuclear envelop breakdown, spindle attachment, etc.) that sometimes involve association with chromatin or the spindle poles during cell division (Capalbo et al., 2011; Hehnly and Doxsey, 2014; Serio et al., 2011). However, FGFR::VENUS does not have a similar chromatin-associated distribution, Additionally, throughout the manuscript, data pertaining to the CLIP::RAB proteins is presented only as overlap between that CLIP-tagged RAB and FGFR::VENUS and our analysis focuses on stage specific shifts in co-localization rather than RAB enrichment at a specific stage. Thus, apparent localization of the RAB constructs to chromatin-enriched regions does not compromise co-localization analysis or the interpretation of our data.

-Fig 4C. I think a control with HALO::Vam2[421-841] without Roscovitine would be useful (it will probably enhance the effect).

Although we agree that including this assay might be useful and might enhance the effect, we don’t consider it a critical assay as our support for this hypothesis is very strong. No similar impact was observed for the other Rabs that we analyzed (which also included the Vam2 dominant negative construct) so the result is unlikely to reflect an artifact due to the impact of the dominant negative construct. Critically, we followed up with extensive data regarding CDK1 dependent phosphorylation of Rab4 and the phosphor-mutants providing extensive additional data in support of the hypothesized role for CDK1 in inhibiting fast recycling during mitotic entry.

-The inhibitor treatment section of the methods refers to a Brefeldin A treatment, but I could not find any reference to it in the text or the figures. Also, I think that 10ug/mL of VX680 corresponds to 21uM not 10uM as stated in the legends of Fig 3 and 5 (MW 464.59).

Thank you for catching these errors, the manuscript has been edited to reflect this correction and remove the reference to Brefeldin A treatment.

Reviewer #4: Review Cota et al
General comments
As cells enter mitosis and round-up the total amount of plasma membrane is reduced through a process that prevents recycling of endosomes back to the plasm membrane. Recycling occurs either via molecularly distinct fast or slow pathways. How these two recycling pathways are shut off during mitosis is currently unknown.
Here the authors nicely demonstrate that the major kinase that drives cells into mitosis (CDK1) prevents fast endosome recycling. In addition, the authors demonstrate that CDK1 also prevents lysosomal degradation of internalized FGF receptors, leading to their accumulation within the cell. To supplement this analysis the authors also show that Aurora kinase (another kinase active in mitosis) prevents the slow recycling pathway. These two kinases thus lead to the accumulation of endocytosed FGF receptors on internal endosomes, allowing them to be recycled to the plasma membrane once the cell exits mitosis. Intriguingly, it is also suggested that the subcellular distribution of internalized FGF receptors is controlled by Aurora kinase, and that this has implications for the cell fate outcome of the two daughter cells (either cranial-cardiac or tail muscle: FoxF is used as an indicator of heart progenitor fate).
This is a well-written article that addresses a fundamental topic using both pharmacological and molecular tools combined with sophisticated volumetric image analysis of fluorescent reporter constructs targeted via transgenesis to the specific cells. I support publication of this article following some minor revision.

One general comment is related to the previous article published by the group (Cota and Davidson, 2015), where they nicely demonstrated that FGF receptors were degraded in mitosis (reversed by MG132 mitotic block) when cell adhesion was inhibited with RapS17N. I therefore expected an update on how lack of adhesion leads to receptor degradation during mitosis given the current finding that CDK1 also shuts off the FGF receptor degradation machinery during mitosis.

As mentioned in response to a previous comment by Reviewer 1, this manuscript is focused on providing an initial characterization of mitotic trafficking, beginning to address this fundamental gap in cell biology, rather than focusing on heart progenitor cell fate induction and the role of adhesion in this process. However, we are very excited to explore how adhesion may bias trafficking as we discuss in our conclusion. In particular, we suggest that adhesion may bias degradation as it resumes during mitotic exit (Figure 6). We have also added some additional language to our discussion regarding this point (see below).

Figures.
Figure 1. FGF receptor moved from plasma membrane to internal stores then back to plasma during mitotic entry, mitosis and mitotic exit respectively.
This figure was fine.

Figure 2. Dynamics of FGFR cycling and association with RAB7 or RAB11 endosomes
Late endosome RAB7, slow recycling endosome RAB11 - OK
Prophase: FGFR and RAB11 peripheral co-localization increased = Slow Cycling Endosomes - OK
Metaphase: FGFR and RAB7 co-localization increased (deep cell Maturation Pathway), FGFR and RAB11 co-localization remained unchanged - OK
Anaphase: FGFR and RAB7 co-localization decreased (recycled or degraded), FGFR and RAB11 peripheral co-localization increased (and returned to plasma membrane) - OK

Figure 3. CDK1 suppresses FGFR degradation during mitotic entry
3A,B. Blocking cells entering mitosis with Roscovitine led to decrease in FGFR staining.
3C. Blocking cells entering mitosis with VX-680 (Aurora kinase inhibitor) did not reduce FGFR staining.
3F-H. E-Cadherin staining not affected by Roscovitine.
Conclusion: CDK1 stabilizes a subset of membrane proteins
Hypothesis: CDK1 suppresses FGFR lysosomal degradation.
3D. Blocking lysosomal degradation with Vam2 expression rescued FGFR signal loss induced by Roscovitane - Nice
3I and Figure S4. A stabilized form of cyclin B (delta 90) that causes sustained CDK1 activity increased FGFR staining - consistent and opposite to Roscovitine finding.
However, the quantification of the delta 90 experiment is missing. Please add.

As indicated in our response to previous Reviewers’ comments (see above), visualization of FGFR::VENUS in founder cells requires antibody staining (Cota et al., 2015) and therefore cannot be imaged or quantified in live cells.

Conclusion: CDK1 activity suppresses RAB4 fast recycling and promotes accumulation of internalized FGFR by also preventing FGFR destruction.
Thus, inhibiting CDK 1 activity should lead to more plasma membrane or peripheral FGFR if FGFR destruction is also blocked (with for example MG132).
Authors were unable to test this because inhibiting CDK1 also prevents FGFR destruction
However, the authors could have combined Roscovitine with MG132 to test this prediction. This experiment may be worth doing of possible.

As a membrane protein FGFR is predominantly degraded using lysosomes rather than the proteasome, inhibition of degradation using the VAM dominant negative construct was the appropriate approach to block degradation. Also, as stated by the Reviewer “inhibiting CDK 1 activity should lead to more plasma membrane or peripheral FGFR if FGFR destruction is also blocked”. Indeed, this is what we observe in founder cells in which CDK1 activity has been inhibited (Roscovitine) and lysosomal degradation has been blocked (Vam2DN expression) (Figure S5).

Figure 4. CDK1 phosphorylation of RAB4 suppresses cycling
Phosphorylation site on RAB4 conserved - interesting that the proline is not conserved.
Phospho-mimetic RAB4 (inactive mutant S199/A199) drove enrichment of FGFR on PM and reduction in peripheral cytoplasm during prophase - nice.
Phospho-mimetic RAB4 (active mutant S199/D199) blocked FGFR recycling during anaphase - nice.
Conclusion: CDK1 phosphorylation of RAB4 inhibits fast receptor recycling during mitosis.
Note - in the cartoon it would appear that blocking Rab4 phosphorylation (S199A) should lead to accumulation of Rab4 vesicles in the periphery on slow recycling vesicles. However, the data indicates that Rab4 is higher on the plasma membrane. Please provide cartoon that displays the data provided in Figures 4H and K. Also, I was not clear why there was not more Rab4 recycled to the plasma membrane via the fast recycling pathway.

This is a good question. It’s important to keep in mind that we are showing FGFR co-localization data for Rab4 and the other Rabs rather than Rab localization data. We have modified the model in Figure 4 and our explanation in the text (changes indicated by red font below) to better explain and illustrate our reasoning. In particular, we show that Rab4 is shed as fast recycling vesicles separate from early endosomes for delivery of their cargo to the plasma membrane. Thus, if fast recycling is inhibited this would lead to increased levels of Rab4/FGFR colocalization.

“This result suggests that CDK1 activity disrupts the delivery of FGFR-enriched fast recycling endosomes to the plasma membrane along with the shedding of Rab4 which occurs during this process (Figure 4D, Ward et al., 2005).”

Figure 5. Aurora kinase regulates maturation and recycling of FGFR endosomes.
VX-680 increased FGFR on plasma membrane - OK.
Hypothesis: Aurora kinase blocks exocytosis of RAB11/FGFR endosomes during mitotic entry.
Aurora kinas inhibition blocked co-localization of RAB11 and FGFR.
Interestingly, VX-680 also decreased RAB7/FGFR co-localization in plasma membrane/peripheral regions.
This result suggests that Aurora kinase promotes maturation of FGFR enriched endosomes.
Hypothesis: this late endosomal pool is important for ventral polarization via biased degradation on founder cell's dorsal side.
In line with this hypothesis, VX-680 gave increased FGFR on founder cell's dorsal side.

Figure 6. Model
OK, although large solid arrows appear unchanged throughout.

Suggestion
Is this specific for FGFR. Could test with Calveolin as previously, for example with D90 does Calveolin behave the same as FGFR?

As noted in the current manuscript, the mitotic trafficking we observe for FGFR does appear to be at least somewhat specific to this receptor as E-Cadherin is not subject to CDK1 dependent suppression of degradation as observed for FGFR (Figure 3F-H). However, we hypothesize that CDK1-dependent suppression of fast recycling may be universal due to direct regulation of Rab4. **We have clarified these aspects of our interpretation in the discussion using the following language.**

Our data also suggests that the previously characterized CDK1-dependent phosphorylation of RAB4 (van der Sluijs et al., 1992) directly suppresses RAB4-mediated recycling during mitotic entry (Figure 4E-K). “This regulatory relationship appears to be broadly conserved as indicted by sequence conservation of the CDK1 phosphorylation site across a wide range of vertebrate Rab4a genes, along with orthologous genes from a variety of tunicates (included *Ciona*, as shown above) amphioxus, echinoderms, mollusks, cnidarians and even potentially in slime molds (data not shown), Interestingly, this phosphorylation site does not appear to be conserved in vertebrate Rab4b genes although they do contain a potential alternate CDK1 target site. We also hypothesis that CDK-1 dependent suppression of fast recycling may be a general feature of mitotic entry deployed in a wide variety of cell types to reduce membrane surface area during mitotic rounding that leads, incidentally, to storage and sequestration of associated membrane proteins. Indeed, this hypothesis aligns with previous data showing that cell rounding involves reduced recycling rates specific to mitotic entry (Boucrot and Kirchhausen 2007).”

Discussion
Good, although I was left wondering about the relative contributions of adhesion-dependent suppression of FGFRs during mitosis and CDK1-dependent suppression of FGFRs during mitosis, and also how loss of adhesion bypasses the CDK1 suppression of FGFR degradation.

This is an important question and a critical focus of our current research. **We have added the following language to the discussion to elaborate more on our model regarding a potential adhesion dependent bias in mitotic trafficking.**

“Our current model posits that integrin-dependent enrichment of caveolin within adhesive membranes dictates polarized FGFR trafficking (Cota and Davidson, 2015). Current studies are focused on determining the specific contributions of integrin and caveolin to this process. We are also investigating whether suppresses FGFR degradation or promotes FGFR recycling, thereby biasing the redistribution of “stored” FGFR during mitotic exit (Figure 6). “

Minor comments
Page 3, Line 3 … for promoting exit specific… Should Be …for promoting exit from specific…
Fig. S5A. Two scale bars appear on same image.
Roscovitine or Roscovatine. Please change all to Roscovitine in the text.

The manuscript has been edited to include these corrections.
