# Supplementary material for: Cyclin-dependent Kinase 1 and Aurora Kinase choreograph mitotic storage and redistribution of a growth factor receptor
Source: PLoS Biol. 2021 Jan 4;19(1):e3001029. doi: 10.1371/journal.pbio.3001029 (PMC7808676; doi:10.1371/journal.pbio.3001029)
Supplement: Attachment — Submitted filename: Response to reviewer.docx [file pbio.3001029.s022.docx]

Response to reviewer’s concerns.

We appreciate the editor’s support and agree that the two concerns raised by Reviewer #1 are clearly beyond the scope of the current manuscript. We do not feel that this reviewer’s assertion that just because we are studying a question regarding cell biology we are required perform cell culture experiments is justified. We have begun to explore this process using a whole embryo assay because mitotic regulation of trafficking appears to be heavily modulated by the in vivo environment. Although we have initiated mammalian cell culture experiments, these extrinsic factors (including the extent of cell-cell or cell -substrate adhesion) will be difficult to control and we do not anticipate having substantial data from this approach for another year. The addition of this data will represent an important follow up extension of the findings documented in the current manuscript.

In response to the third reviewer’s suggestion we have added a control image to figure three, illustrating the lack of discernable FGFR::Venus staining in live embryos under regular conditions. Because there is no signal from FGFR::Venus in these embryos, we co-electroporated with Mesp>H2B::RFP allowing us to identify transgenic, mitotic heart founder lineage cells. As is clear in the control image, these is no staining in the GFP/YFP channel above background levels.

We also changed the legend for Figure 1 as requested which now reads - “Some regions are labeled with an a or b to denote significant changes (p<0.05) that occurred within this region across stages. Other regions are labeled n.s. to denote that no significant changes occurred for the indicated stages.”
